# Phlegmasia cerulea dolens of the upper extremity treated with anticoagulation and leech therapy

**DOI:** 10.1016/j.jvscit.2023.101296

**Published:** 2023-08-18

**Authors:** Julian Melchor, Kenneth Leong, Jeffrey B. Edwards

**Affiliations:** a Sarasota Regional Medical School Campus, Florida State University College of Medicine, Sarasota, FL; b Department of Plastic Surgery, Sarasota Memorial Hospital, Sarasota, FL; c Department of Vascular Surgery, Sarasota Vascular Specialists, Sarasota, FL

**Keywords:** Deep vein thrombosis, Phlegmsia, Phlegmasia cerulea dolens, Leech therapy, Upper extremity

## Abstract

Phlegmasia cerulea dolens is a serious manifestation of venous thrombosis that has a high risk of morbidity and mortality. If not promptly treated on presentation, progressive extremity ischemia and potential gangrene can lead to emergency amputation of the affected limb. Most commonly, the lower extremities are affected, and reports of upper extremity phlegmasia are scarce. We report the successful treatment of phlegmasia cerulea dolens of the distal upper extremity using leech therapy combined with anticoagulation.

Phlegmasia is a rare, limb-threatening manifestation of deep vein thrombosis.^1^ Although the exact incidence is unknown, phlegmasia cerulea dolens and phlegmasia alba dolens are associated with high morbidity and mortality if not promptly treated.^2^ Phlegmasia is most reported in the lower extremities, and reports of upper extremity involvement are scarce.^1^ Progression to arterial ischemia and venous gangrene can lead to amputation.^1^^,^^2^ Although no guidelines are available for phlegmasia treatment, rapid therapy is necessary and should be aimed at alleviating venous obstruction to prevent progression to gangrene. Several studies have reported that leech therapy has the potential to treat venous congestion and thrombosis by enhancing blood and lymphatic flow.^3^ Although leech therapy was used for bloodletting in early human times, it has also been used to reduce venous congestion during plastic and reconstruction surgery.^4^ The successful use of leech therapy within these surgical specialties displays promising application for the field of vascular surgery. We report the case of an 81-year-old woman who presented with an acute abdomen due to an incarcerated hernia and right upper extremity phlegmasia cerulea dolens that was treated successfully with anticoagulation and leech therapy. The present patient provided verbal and written informed consent for the report of her case details and imaging studies.

## Case report

An 81-year-old active woman presented with small bowel obstruction. An initial trial of conservative management failed, and exploratory laparotomy was recommended. She experienced vasovagal syncope shortly after presentation, and advanced cardiovascular life support was briefly performed. Vascular surgery consultation was requested before surgery because of the profound cyanosis of her right hand and distal forearm. She reported paresthesia and numbness of all digits. A right dorsal forearm intravenous line had been removed.

Her pertinent medical history included hormone replacement therapy. Testing for COVID-19 (coronavirus disease 2019) was negative on admission. On physical examination, she was hypotensive, and her right hand was cyanotic from above the wrist and extending to the fingertips. Triphasic Doppler signals were detected in the radial, ulnar, palmar arch, and digital arteries. Her superficial veins appeared thrombosed. Venous duplex ultrasound confirmed thrombosis of all superficial and deep venous structures in the forearm, sparing the basilic and cephalic veins above the elbow and the axillary and subclavian veins. Arterial duplex ultrasound demonstrated patent upper extremity arteries. Anticoagulation and arm elevation were recommended; however, she was taken for exploratory laparotomy, during which an incarcerated hernia was found and 80 cm of small bowel resected. Immediately after surgery, her right hand cyanosis had progressed, and staccato radial and ulnar artery Doppler signals were present. The right hand had tense edema. Anticoagulation with intravenous heparin titrated to maintain an activated partial thromboplastin time of 60 to 80 seconds and arm elevation were initiated. Thrombolysis was contraindicated because of her recent surgery. Owing to involvement of the distal forearm and hand, consultation with plastic and microvascular hand surgery was sought, and a trial of leech therapy was recommended and initiated. Fasciotomy was discussed; however, in the absence of clinical compartment syndrome, this was not indicated.

After 12 hours of leech therapy, in addition to anticoagulation and arm elevation, profound improvement had occurred in venous congestion, with a return of palpable radial and ulnar pulses. The patient's clinical manifestation of numbness and decreased motor function had also improved. She was seen in consultation with a hematologist who believed it was provoked in the context of estrogen replacement therapy. The therapies were continued, and leech therapy was slowly weaned off over ∼3 weeks, as informed by clinical experience and decisions of the plastic and microvascular hand surgeon, at which point she had complete resolution of edema with no detectable sensory or motor deficits. Blood loss secondary to leech therapy was notable, and a total of 14 U of packed red blood cells was administered during the 3 weeks of inpatient leech therapy. No other sources of blood loss were detectable at this time.

At the completion of leech therapy, repeat duplex ultrasound confirmed normal arterial flow and partial recanalization of the radial and ulnar veins. She was discharged home with apixaban for 3 months. At her 3-month follow-up, duplex ultrasound showed chronic thrombophlebitic changes, and she had complete preservation of motor and sensory function in the affected hand.

## Discussion

Phlegmasia is a rare manifestation of venous thrombosis affecting the superficial and deep veins.^1^ This limb-threatening condition has a high rate of morbidity and mortality if not promptly treated.^2^ Most reported cases have occurred in the lower extremity, with only a few cases demonstrating upper extremity involvement. The diagnosis is challenging and can be confused with other ischemic disorders.^1^ Without prompt treatment, the resulting venous congestion and arterial ischemia can progress to tissue gangrene, limb amputation, and, even, death.^1^^,^^2^ Currently, no guidelines are available for the management and treatment of phlegmasia; however, a consensus exists that prompt treatment is necessary.^5^

A classic triad to help diagnose phlegmasia cerulea dolens is limb swelling, acute ischemic pain, and cyanosis.^5^ Cyanosis is a major pathognomonic feature of phlegmasia.^1^ These symptoms can then progress to venous congestion, ischemic gangrene, circulatory collapse, and shock that can eventually lead to death.^1^^,^^5^ Because of this sequela of possible life-threatening symptoms, it is crucial to recognize the symptoms early in the disease course; 40% to 60% of patients can have a delayed diagnosis of their condition, leading to irreversible venous gangrene of the affected limb.^5^ Thus, treatment at an early, reversible phase is vital to salvage the extremity.

Supportive measures and elevation of the affected limb should be initiated once the diagnosis is suspected.^1^ The goal for the treatment of phlegmasia is to provide effective, rapid therapy to prevent further venous congestion, limb ischemia, and venous gangrene.^2^ The treatment paradigms reported in the literature are anticoagulation, surgical thrombectomy, fasciotomy, catheter-directed thrombolysis, and/or percutaneous transluminal angioplasty.^1^^,^^5^ In some cases, a combination of these therapies must be used, depending on the severity and extent of limb involvement.

We describe effective treatment of phlegmasia cerulea dolens using a combination of systemic anticoagulation and leech therapy. This modality was used because of primary involvement of the distal forearm and hand, believed not to be amenable to open thrombectomy techniques. Because of her recent surgery, thrombolysis was contraindicated. Leech therapy is believed, not only to relieve the venous congestion and thrombolysis in phlegmasia, but also to enhance blood and lymphatic flow and suppress inflammation.^3^ The relief of venous congestion using leech therapy is suspected to result from the physical extraction of blood consumed by the leech and the medicinal properties in leech saliva.^6^ Venous and arterial flow are replenished by the anticoagulative, antiplatelet, and vasodilatory properties of the leech saliva, decreasing thrombi formation and venous congestion.^6^ It is also believed that one of the most abundant anticoagulants in leech saliva, also known as hirudin, even contains neovascularization and angiogenesis promoting properties.^6^

**Fig 1:**
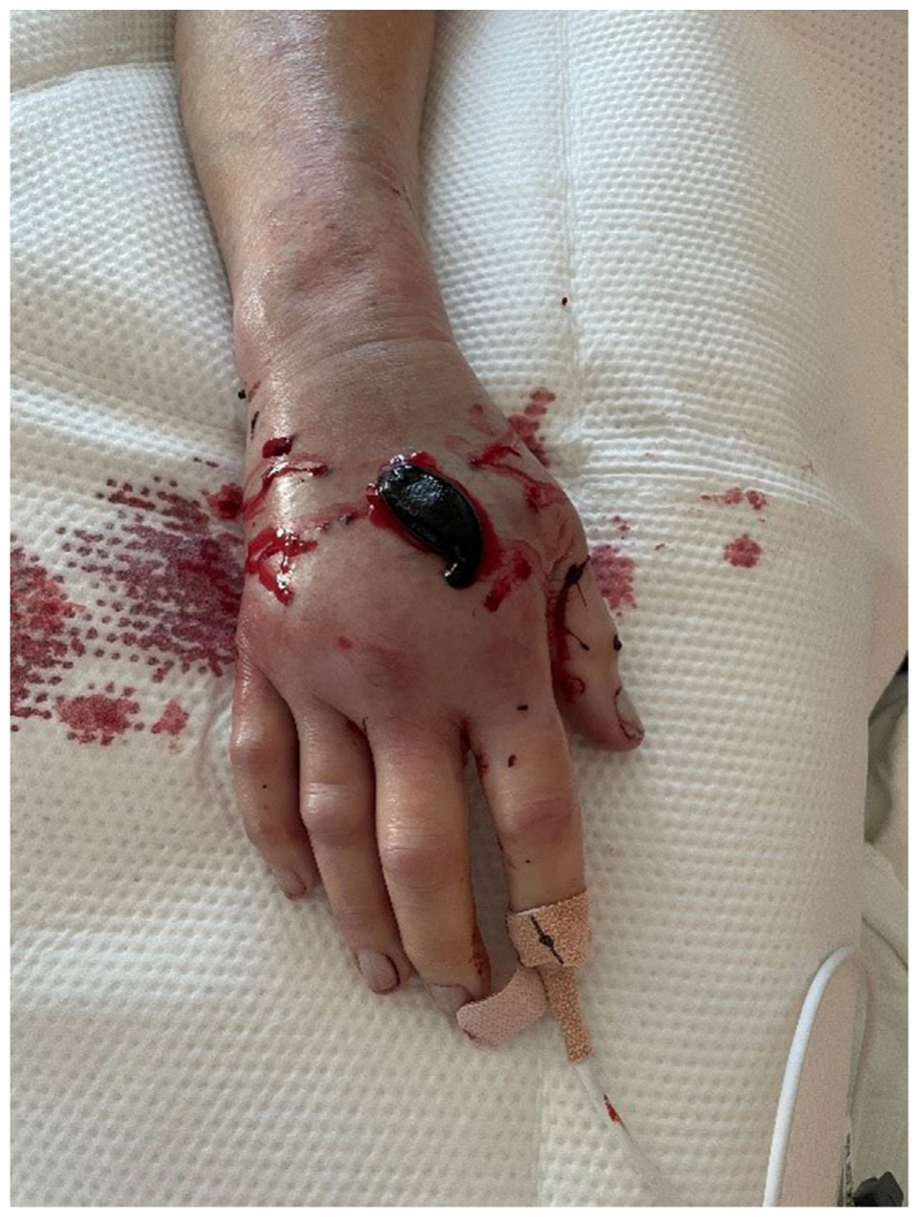
Photograph showing ongoing leech therapy on the dorsal right hand.

**Fig 2:**
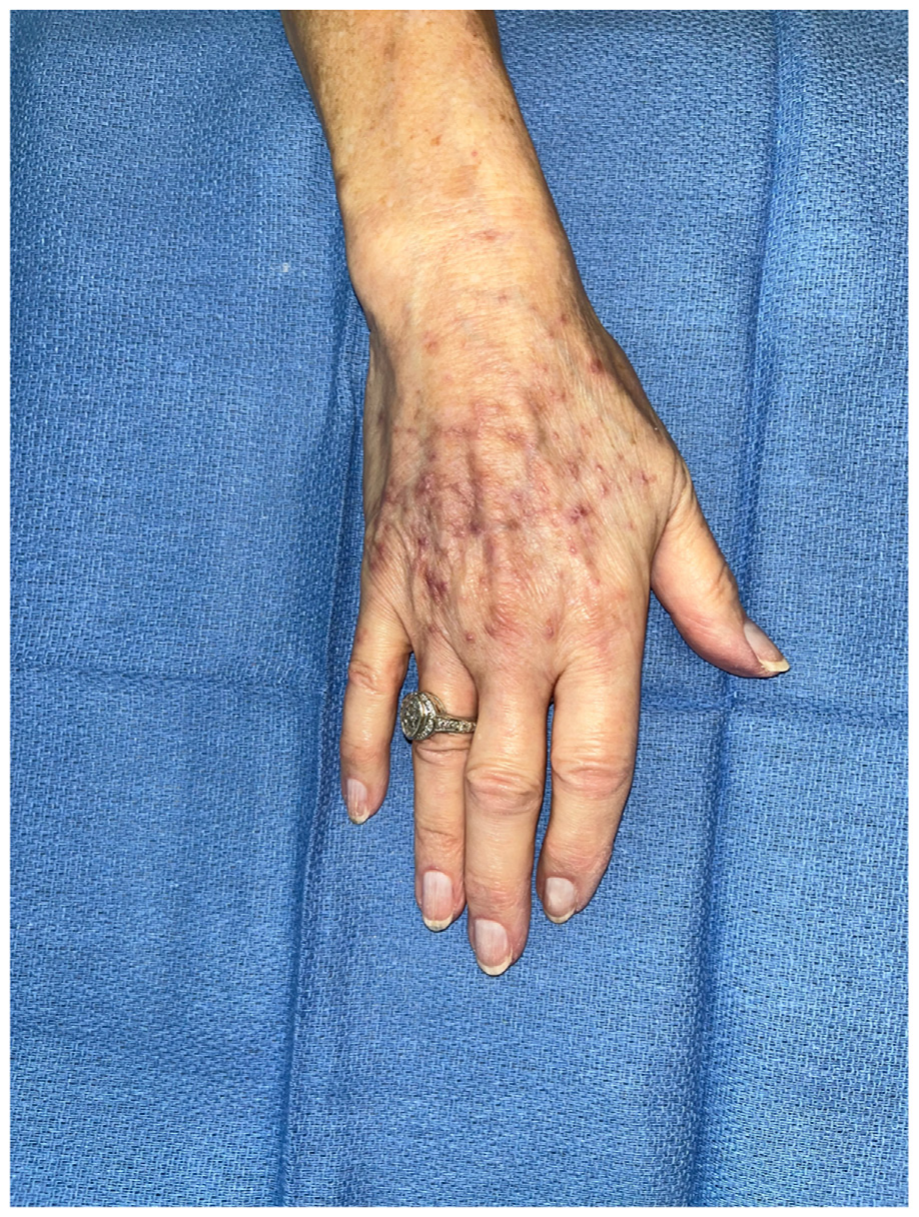
Photograph showing the right upper extremity after leech therapy.

Once supportive measures and elevation of the affected limb have been initiated, our case demonstrates that additional management such as anticoagulation with leech therapy is a possible successful option for upper extremity phlegmasia cerulea dolens. Our patient underwent 3 weeks of leech therapy with complete resolution of her symptoms by the end of her treatment course. Fig 1 demonstrates our patient undergoing leech therapy with a leech placed on the dorsal right hand. During the middle of her treatment plan, she had missed a few therapy sessions owing to complications regarding the placement and supply of the leeches. This caused a quick recurrence of her primary symptoms of pain, swelling, and paresthesia, which required almost immediate reinitiation of the leeches, with progressive symptom resolution. Fig 2 displays the patient's upper extremity after completion of the 3 weeks of leech therapy. One limitation of this approach is the associated blood loss, which can be significant, as indicated by the requirement for 14 U of red blood cells over the duration of her treatment course.

## Conclusions

Phlegmasia cerulea dolens is an uncommon manifestation of deep vein thrombosis mainly involving the lower extremity. With minimal reports of upper extremity phlegmasia, it is important to promptly recognize the symptoms and treat the condition. Our case is unique in that it demonstrates phlegmasia isolated to the forearm and hand that was successfully treated with anticoagulation and leech therapy.
